# Zinc fever in a painter and varnisher: a case report

**DOI:** 10.1186/s13256-024-04651-8

**Published:** 2024-07-10

**Authors:** Kerstin Belting, Christian Eisenhawer, Rolf Merget, Thomas Brüning, Christian Monsé

**Affiliations:** Institute for Prevention and Occupational Medicine of the German Social Accident Insurance, Institute of the Ruhr-Universität Bochum (IPA), Bürkle-de-la-Camp-Platz 1, 44789 Bochum, Germany

**Keywords:** Low temperature, Exposure to zinc oxide, Occupational accidents, Painter, Zinc fever, Varnisher, Painter, Aerosol

## Abstract

**Background:**

Zinc fever is well described in medical literature, particularly in workers after handling zinc-containing materials at high temperatures e.g., in the welding of hot-dip galvanized steel sheets. It is not known whether zinc fever also occurs at low temperatures.

**Case presentation:**

We present the case of a 33-year-old Caucasian atopic painter and varnisher with work-related dyspnea, sweating, as well as multiple occurrences of fever. He was sent to Institute for Prevention and Occupational medicine of the German Social Accident Insurance, Institute of the Ruhr-Universität Bochum (IPA) for the evaluation of isocyanate asthma, but an inhalative challenge with hexamethylene diisocyanate was negative. Since symptoms were closely related to the use of zinc coatings at room temperature without adequate protective measures, the diagnosis of zinc fever was made. After exposure cessation the worker immediately became symptom-free. The work as painter and varnisher may be associated with various exposures to hazardous substances. Besides solvents, epoxy compounds and isocyanates, which can cause obstructive respiratory diseases; additionally, zinc-containing agents should be considered as health hazards.

**Conclusions:**

This case demonstrates that zinc fever may occur also after application of zinc coatings by spray painting at low temperatures.

## Background

Zinc fever is an occupational disease caused by the inhalation of finely dispersed particles that form when certain metals volatilize.

When zinc or one of its alloys is burnt, melted, or heated in air to about 500 °C zinc oxide is generated in form of an ultra-fine aerosol, which grows into larger agglomerates over time [1]. Inhalation of these particles can cause metal fume fever. In the past, symptoms of this disease were observed in industrial workers engaged in burnishing, galvanising, welding zinc, or galvanised iron [2].

Zinc fever is accompanied by flu-like symptoms that occur shortly after these activities. An improvement occurs over the course of the working week. On re-exposure after returning to the workplace, symptoms, such as fever, malaise, muscle aches, and pains, and a feeling of being unwell, recur. For this reason, the disease is sometimes referred to as “Monday fever.” The first cases were reported in the 1830s [3, 4].

The syndrome is well described in medical literature, particularly in workers after handling zinc containing materials at high temperatures e.g., in the welding of hot-dip galvanized steel sheets. It is not known whether zinc fever occurs at low temperatures.

## Case presentation

A 33-year-old Caucasian male painter and varnisher was sent to the Institute for Prevention and Occupational medicine of the German Social Accident Insurance, Institute of the Ruhr-Universität Bochum (IPA) in December 2022 to perform an inhalative challenge test with diisocyanates. After his apprenticeship as a painter and varnisher from 2006 to 2009 he worked mostly as a spray painter in several companies. In his last job he worked as a spray painter from July 2020 to December 2021. He was a smoker, but healthy until June 2021 when he developed work-related dyspnea, chest tightness, “flu-like” symptoms and multiple fever episodes in the evenings of work days. He was dismissed by his employer owing to repetitive sick leaves. There were no exhaust systems, and painting was carried out in a hall without spatial separation. The personal protective equipment consisted of a half mask, the filter of which was not changed regularly.

After exposure cessation he immediately became symptom-free. A technical evaluation of the worker’s exposures by the technical service of the accident insurance yielded exposures to solvents, epoxy resins, zinc oxide-containing (ZnO) coatings, and diisocyanates, mostly hexamethylene diisocyanate (HDI). The technical report and the anamnestic statements of the patient mentioned that the working conditions were inadequate. The worker gave detailed information of his working conditions. His main tasks were spray painting of large machine parts. The zinc coating was applied at cold temperatures. He had partly worked in the “zinc mist” without a mask, as the half mask could no longer be used owing to increased breathing resistance. After a vacation without symptoms he experienced several reproducible episodes of respiratory irritation, sweating, a feeling of pressure in the lungs, shortness of breath, and fever of up to 40 °C when resuming work.

The medical examinations included a physical examination with comprehensive otorhinolaryngeal examination as well as an electrocardiogram (ECG) and blood sampling with determination of blood count, differential blood count, inflammatory parameters, transaminases, protein electrophoresis, and urine tests. Lung function was recorded.

The medical examinations, including otolaryngologic examinations, were unremarkable. The differential blood count showed a neutrophilia of 9/nL, but laboratory chemistry and urine examinations yielded normal values. The electrocardiogram (Mortara ELI280, Milwaukee, USA) was unremarkable. Lung function analysis using body plethysmography [5] and spirometry [6] in a linked maneuver with a SpiroScout bodyplethysmograph (Ganshorn, Niederlauer, Germany) could not confirm the presence of airway obstruction. Methacholine testing was performed with Provo.X B. (Ganshorn, Niederlauer, Germany) with a nebulizer output of 0.436 ml/minute with a particle size range between 1 and 5 µm in an American Thoracic Society (ATS) [7] dose-adapted, 1-concentration, 4-step test. Taking into account the guidelines of the American Thoracic Society and European Respiratory Society [7, 8] Bronchial hyperresponsiveness was not detected. Fractional exhaled nitric oxide (FeNO) was measured using a portable electrochemical analyzer (NIOX Vero, Circassia, Bad Homburg, Germany). FeNO was 11 ppb (within reference values). Skin prick testing was performed using commercial skin prick test solutions (SPT) from various manufacturers. Measurements of total immunoglobulin E (IgE) and specific IgE antibodies to house dust mites (d1 and d2), mold mix (mx1), hexamethylene diisocyanate (HDI, k77), toluene-2,4-disocyanate (TDI, k 75), and methylene diphenylisocyanate (MDI, k76) were performed using commercial ImmunoCAPs from Thermo Fisher Scientific (Waltham, USA) SPTs to the house dust mites *Dermatophagoides pteronyssinus* and *Der*. *farinae* showed wheal diameters of 4 mm and 5 mm, respectively. Total IgE was 14 kU/L. Specific IgE antibodies to both mites were detected (CAP class 2), but not to diisocyanates. To exclude isocyanate asthma, a standardized inhalation challenge with HDI was performed. Inhalation testing with HDI was performed in five different exposure levels. A blank measurement was followed by exposures to 3 ppb for 10 minutes, 5 ppb for 10 minutes, 10 ppb for 10 minutes, 10 ppb for 20 minutes, and 10 ppb for 40 minutes [9]. Lung function measurements were performed after each exposure level, as well as half, two, four, and 24 hours post exposure, but airway obstruction could not be demonstrated.

As part of the medical history, questions regarding zinc fever were elicited using a questionnaire previously used in the study on concentration-dependent effects of inhaled ZnO aerosols by Monsé *et al*. 2018 [10].

The questionnaire was retrospectively completed by the worker for three time points in 2021 when he returned to work after a holiday or after sick leave.

A detailed list of events is shown in the timeline below (Table 1). The worker’s symptoms are listed according to their severity (Table 2). It is evident that the worker reported the typical “flu-like” symptoms (including muscle pain, chills, feeling sick, feeling unwell, feeling feverish, feeling cold, shortness of breath, headache, fatigue, nausea, and a metallic taste in the mouth) as described for zinc fever. Atypical symptoms, such as watery/burning/dry/itchy eyes, abdominal pain, or itchy skin, were not reported.

**Table 1:** Timeline of events

Date	Complaints	Other	Personal protective equipment
T0		Start of the employment relationship	Ventilated helmet with independent air supply
T0–T1		Ventilated helmet with independent air supply breaks down	Half mask with screw-on filter. Changing the filters is becoming increasingly rare, sometimes working without a mask
T1	Regular chills on days off		
T2	Symptom free	3 weeks holiday	
T3	Irritation of the respiratory tract	Return to work after holiday	
T4	Sweating, dyspnea, feeling of pressure, general feeling of illness	Working	
T5	Same Symptoms	Working	
T6	Fever 40 °C	Sick note for 3 weeks	
T7	Sweating, dyspnea	Return to work complaints after 3 hours	
T8		Sick note for 3 days	
T9	Sweating, dyspnea	Sick note	
T10		Dismissal	
T11	Examination at IPA: T10+		

T0: start of employment relationship

T1: T0 + 10 months

T2: T1 + 3 months

T3: T2 + 1 day

T4: T3 + 1 day

T5: T4 + 1 day

T6: T5 + 1 day

T7: T6 + 3 weeks

T8: T7 + 1 day

T9: T8 + 5 days (including weekend)

T10: T9 + 2 weeks

T11: T10 + 13 months

**Table 2:** Symptoms of the worker after he had returned to work after a holiday (T1) or periods of sick leave (T7 and T9)

	Severity of symptoms (0–6 points)		
	T3	T7	T9
Fatigue	4	4	4
Itchy eyes	1	0	0
Abdominal pain	0	0	0
Metallic taste in mouth	4	4	4
Dry nose	0	0	0
Throat irritation	0	0	0
Headache	6	5	5
Cough	1	1	1
Burning eyes	0	0	0
Feeling warm	5	0	0
Itchy skin	0	0	0
Nausea	4	2	2
Shortness of breath	6	2	2
Dry eyes	0	0	0
Sweating	5	3	3
Irritation of the eyes	0	0	0
General irritability	0	0	0
Feeling of fever	6	3	3
Feeling cold	5	2	2
Chills	6	3	3
Runny nose	2	0	0
Muscle pain	4	3	3
Feeling unwell	4	3	3
Watering eyes	0	0	0
Feeling sick	5	3	3
Muscle cramps	3	1	1

Scale of 0–6 (0 = not at all, 6 = very strong)

On the basis of the exposure to Zn-containing aerosols and a typical medical history a diagnosis of metal fume fever was made. The diagnosis is corroborated by complete immediate recovery of the worker’s symptoms after exposure cessation. Asymptomatic sensitizations to house dust mites were detected by SPT and CAP, but preventive measures or therapy were not recommended.

In a previous job, the worker had already sprayed zinc coatings. Appropriate protective masks were available, and filters were changed several times a day. Under these conditions “flu-like” symptoms or respiratory complaints did not occur.

## Discussion

The worker’s diagnosis of zinc fever is based on typical symptoms, which develop a few hours to 48 hours after exposure, such as chills (“flu-like” symptoms), fever, muscle and joint pain, circulatory disorders, nausea and vomiting, cough, chest pain, and respiratory complaints [11, 12]. Zinc fever is particularly known to develop during the welding of zinc-coated materials. The highly heated zinc oxidizes in air to ZnO and can be released into the environment as a zinc-containing aerosol. Inhaled ZnO causes systemic and local effects in the organism [10, 13, 14]. The course of the symptoms with occurrence after periods away from work (Monday fever) and immediate recovery after exposure cessation are compatible with this diagnosis. Although “flu-like” symptoms and fever are atypical for isocyanate asthma, hypersensitivity pneumonitis has been reported after diisocyanate exposure [15]. Whereas asthma could not be shown in this case, hypersensitivity pneumonitis is not probable because Monday fever and immediate complete recovery after exposure cessation argue against this diagnosis. Although we did not favor the diagnosis of isocyanate hypersensitivity pneumonitis, we performed inhalation testing to exclude it. The negative result of the inhalation challenge allows us to exclude isocyanate asthma or hypersensitivity pneumonitis with sufficient certainty. Zinc fever is well described in welders. In painters, literature search revealed no reported cases. When ZnO is inhaled as an aerosol, it is partially deposited in the respiratory tract. In the alveoli, the particles are taken up and decomposed by macrophages for elimination from the lungs. ZnO dissolves owing to a decrease of pH, releasing zinc ions in locally high concentrations. These cause the systemic and local effects that may eventually become clinically apparent as zinc fever [16]. When inhaling ZnO aerosols, from a toxicological-mechanistic point of view, it makes little difference whether this substance is produced as a waste product by thermal processes or is released during processing at room temperature [14]. Inhaled concentrations of zinc oxide as low as 1 mg/m^3^ over 4 hours can lead to an increase in inflammatory markers in the blood C-reactive protein (CRP), serum amyloid A (SAA), and neutrophil granulocytes. In addition, at concentrations of 2 mg/m^3^ over 4 hours, “flu-like” symptoms, such as fever and nausea, may occur [10]. The Maximum workplace concentration (MAK) value, defined by the German Research Foundation Senate Commission, for zinc is 2 mg/m^3^ and refers to the inhalable fraction (E-fraction). The MAK value of 0.1 mg/m^3^ zinc refers to the alveolar fraction (A-fraction), but to date is not a legally binding occupational exposure limit in Germany [17]. A new occupational exposure limit value is currently being discussed by the regulatory authorities. Measurement of airborne ZnO at the worker’s workplace were not available. Owing to the worker’s description of poor working conditions it can be assumed that he was exposed to high concentrations of ZnO. He had been exposed to ZnO earlier in another workshop with better working conditions without zinc fever. Also the latency of almost a year between the beginning of ZnO exposure and symptoms, which can be explained by less frequent changes of the filters of his respiratory protection during later time points of his occupation argues for a clear dose–response relationship and, thus the effectiveness of preventive measures. It is not known whether further workers of his workshop developed zinc fever. As the worker had been dismissed, preventive measures were recommended to protect his colleagues.

In the worker’s case, several episodes of an occupational accident were overlooked. Dismissal was possibly unlawful. To prevent further cases in the future, it must firstly be recognized that zinc fever can also occur at low temperatures. Then either a substitution of the zinc or technical, organizational measures should be implemented or sufficient personal protective equipment should be provided by the company, whereby the substitution or technical measures are of the greatest importance. These measures should be regularly reviewed by the employers’ liability insurance association.

## Conclusion

The occurrence of zinc fever is usually associated with the handling of galvanized components under high temperatures, such as welding.

The present case shows that the same symptoms can also occur when zinc-containing aerosols are inhaled at room temperature. Therefore, it is important to record the detailed working conditions when assessing occupational diseases or occupational accidents. Possible triggers of zinc fever could otherwise be easily overlooked.

## Data Availability

The clinical documentation of the presented case cannot be made public owing to the detailed identifiable information of the patient.

## Abbreviations

ATS: American Thoracic Society
CRP: C- reactive-Protein
ECG: Electrocardiogram
E-fraction: Inhalable fraction
FeNO: Fractional exhaled nitric oxide
HDI: Hexamethylene diisocyanate
IPA: Institute for Prevention and Occupational medicine of the German Social Accident Insurance, Institute of the Ruhr-Universität Bochum
MAK: Maximum workplace concentration, defined by the German Research Foundation Senate Commission
MDI: Methylene diphenylisocyanate
SAA: Serum amyloid A
SPT: Skin prick test solutions
TDI: Toluene-2,4-disocyanate
ZnO: Zinc oxide
